# Extreme Heat Exposure is Associated with Lower Learning, General Cognitive Ability, and Memory among US Children

**DOI:** 10.31586/ojn.2025.1277

**Published:** 2025-01-10

**Authors:** Shervin Assari, Hossein Zare

**Affiliations:** 1 Department of Internal Medicine, Charles R. Drew University of Medicine and Science, Los Angeles, CA, United States; 2 Department of Family Medicine, Charles R. Drew University of Medicine and Science, Los Angeles, CA, United States; 3 Department of Urban Public Health, Charles R. Drew University of Medicine and Science, Los Angeles, CA, United States; 4 Marginalization-Related Diminished Returns (MDRs) Center, Los Angeles, CA, United States; 5 Department of Health Policy and Management, Johns Hopkins Bloomberg School of Public Health, Baltimore, MD, United States; 6 School of Business, University of Maryland Global Campus (UMGC), Adelphi, MD, United States

**Keywords:** Extreme Heat, Climate Change, Child Development, Socioeconomic Status, Racial Disparities, Vulnerable Populations, Cognitive Function

## Abstract

**Background.:**

The increasing frequency and intensity of extreme heat exposure is a significant consequence of climate change, with broad public health implications. While many health risks associated with heat exposure are well-documented, less research has focused on its impact on children’s cognitive function.

**Objectives.:**

This study examines the relationship between extreme heat exposure and various domains of cognitive function in children.

**Methods.:**

Data were drawn from the Adolescent Brain Cognitive Development (ABCD) study. Key variables included race/ethnicity, age, gender, family socioeconomic status (SES), heatwave exposure, and multiple cognitive domains: total composite score, fluid composite score, crystallized intelligence, reading ability, picture vocabulary, pattern recognition, card sorting, and list recall. Structural equation modeling (SEM) was used for data analysis.

**Results.:**

A total of 11,878 children were included in the analysis. Findings revealed significant associations between extreme heat exposure and lower cognitive performance across multiple domains. The strongest adjusted effects were observed in pattern recognition (B = −0.064, p < 0.001) and reading ability (B = −0.050, p < 0.001), both within the learning domain, as well as total composite cognitive ability (B = −0.067, p < 0.001), fluid composite (B = −0.053, p < 0.001), and crystallized intelligence (B = −0.061, p < 0.001), all within general cognitive ability. Weaker but still significant associations were found for list recall (B = −0.025, p = 0.006) and card sorting (B = −0.043, p < 0.001) within the memory domain, as well as picture vocabulary (B = −0.025, p = 0.008) within general cognitive ability. These associations remained significant after controlling for demographic factors, race/ethnicity, family SES, and neighborhood SES.

**Conclusions.:**

This study underscores the impact of climate change on cognitive function disparities, particularly in learning and general cognitive ability among children exposed to extreme heat. Findings highlight the need for targeted interventions to mitigate the cognitive risks associated with heat exposure in vulnerable populations.

## 1. Introduction

Global temperatures have reached unprecedented highs over the past decade, marking a dramatic shift in climate patterns since the mid-19th century. The Intergovernmental Panel on Climate Change (IPCC) [1] predicts that the frequency and severity of extreme heat events will continue to increase, largely driven by climate change. These changes have amplified concerns about the far-reaching consequences of extreme weather, particularly for vulnerable populations in low-income areas where resources to manage such environmental challenges are scarce [2]. Economically, extreme heat has been linked to rising costs for businesses, reduced productivity, lower agricultural yields, and increased absenteeism, among other challenges [3–6].

The health implications of extreme heat are equally alarming [7, 8]. High temperatures and heat stress are associated with elevated mortality and morbidity rates, adverse pregnancy outcomes, and deteriorations in mental health. Heat stress can also impair physical work capacity and motor-cognitive performance, leading to declines in productivity and heightened risks of occupational health issues [2]. Over half of the global population, including more than 1 billion workers, is exposed to high heat episodes annually, with significant health repercussions for nearly one-third [2]. However, many of these health risks are preventable through the implementation of heat action plans that incorporate behavioral and biophysical strategies [2].

The enduring nature of extreme heat events has caused an increase in heat-related mortality, a trend expected to worsen as climate change progresses. In tropical regions, rising temperatures may surpass the physiological limits of heat tolerance, posing severe risks to human survival in the coming decades [2]. Factors such as urbanization, population growth, aging, and socioeconomic development further compound the risks associated with heat exposure. Urban areas are particularly vulnerable due to the accumulation of anthropogenic heat from transportation and buildings [2].

Children are among the most vulnerable groups to the adverse effects of extreme heat. Research indicates that high temperatures are associated with increased emergency department visits among children, particularly those aged 0-4 years, with the strongest associations observed on the same day of exposure. Certain subgroups, including children under 1 year of age, exhibit delayed effects, with significant associations appearing up to three days after exposure [9, 10]. Across diagnostic categories, extreme heat has been linked to higher risks of heat-specific diagnoses, general symptoms, infectious diseases, and injuries [9, 10].

The Centers for Disease Control and Prevention (CDC) emphasize that children are especially at risk during heat waves, with potential disruptions to their daily activities and social interactions. These impacts are exacerbated for children living in poverty, who may lack access to mitigating resources such as air conditioning. Despite these significant concerns, the effects of extreme heat on children’s developmental outcomes, including cognitive function across various domains, remain understudied.

This study seeks to address this gap by investigating the relationship between extreme heat exposure and cognitive function in children. Using data from the Adolescent Brain Cognitive Development (ABCD) [16–25] study, we explore whether exposure to extreme heat is associated with variations in cognitive performance, considering potential confounding factors such as neighborhood and family socioeconomic status (SES) [26] and race/ethnicity [48,49,52,53]. This research contributes to a deeper understanding of how climate change may impact youth development and whether neighborhood or family SES can mitigate these effects.

## 2. Methods

### 2.1. Study Design

This study employed a secondary analysis of baseline data collected as part of the Adolescent Brain Cognitive Development (ABCD) study [16–25], a large-scale, longitudinal research initiative designed to examine the developmental trajectories of pre-adolescent children. The ABCD study is characterized by its robust methodology, encompassing a nationally representative cohort with significant racial, ethnic, and socioeconomic diversity. Participants were primarily recruited from schools, with efforts made to ensure broad geographic and demographic representation. Detailed descriptions of the study design, recruitment strategies, and protocols are available in the literature [16–25]. Key strengths of the ABCD dataset include its longitudinal framework, the extensive sample size, and the inclusion of participants from diverse socioeconomic and racial/ethnic backgrounds, making it a valuable resource for studying complex developmental outcomes.

### 2.2. Analytical Sample

The analytical sample for this research included all youth participating in the ABCD study, irrespective of their socioeconomic or racial/ethnic background. At the baseline assessment, participants were between the ages of 9 and 10 years. After applying inclusion criteria, the final sample consisted of 11,878 children, providing substantial statistical power to detect associations and evaluate multivariable relationships. The inclusion of a large and diverse cohort allowed for nuanced analyses of cognitive outcomes across various sociodemographic contexts.

### 2.3. Ethical Considerations

This study adhered to ethical standards for research involving human participants. Approval for the ABCD study was obtained from the Institutional Review Board (IRB) at the University of California, San Diego (UCSD). Informed consent was secured from parents or legal guardians, and assent was obtained from all participating children. Data confidentiality and participant privacy were prioritized throughout the research process, in compliance with relevant ethical guidelines and regulations.

### 2.4. Study Variables

#### Race/Ethnicity:

The racial and ethnic identity of participants was reported by their parents and categorized as non-Latino White (reference group), Black, Latino, Asian, and other/mixed race or ethnicity. This variable was used to examine differences in cognitive outcomes across racial and ethnic groups.

#### Neighborhood Median Home Value:

Economic indicators for participants’ residential areas were derived using zip code data from the ABCD study’s residential history records. Median home value within each participant’s zip code was used as a continuous measure of area-level socioeconomic status (SES), with higher values reflecting more affluent neighborhoods.

#### Family Socioeconomic Status:

Family SES was quantified based on parental education and household income. These variables were treated as continuous measures, with higher scores denoting greater socioeconomic advantage.

#### Cognitive Function:

Cognitive performance was assessed across multiple domains using standardized measures from the NIH Toolbox. These included total composite, fluid composite, crystallized intelligence, reading ability, picture vocabulary, pattern recognition, card sorting, and list recall tasks. Higher scores across these domains indicated better cognitive function. Table 1 shows how NIH Toolbox measures in the ABCD evaluate memory, learning, and general cognitive ability [27–29, 54].

### 2.5. Data Analysis

All analyses were conducted using Stata statistical software. Descriptive statistics, including means and standard deviations (SD), were computed for continuous variables. Bivariate relationships were assessed using Pearson correlation tests to explore associations among variables. Multivariable analyses were performed using Structural Equation Modeling (SEM), a robust approach for evaluating relationships among observed and latent variables. The primary outcomes were scores across various cognitive domains, with heat wave exposure as the main predictor. Covariates included age, gender, race/ethnicity, and family SES, which were treated as potential confounders. Correlations between variables were tested to ensure collinearity did not bias the models, with all pairwise correlations below the threshold of 0.6. The results were presented as standardized path coefficients (β), along with 95% confidence intervals (CIs) and p-values.

## 3. Results

**Figure 1.**
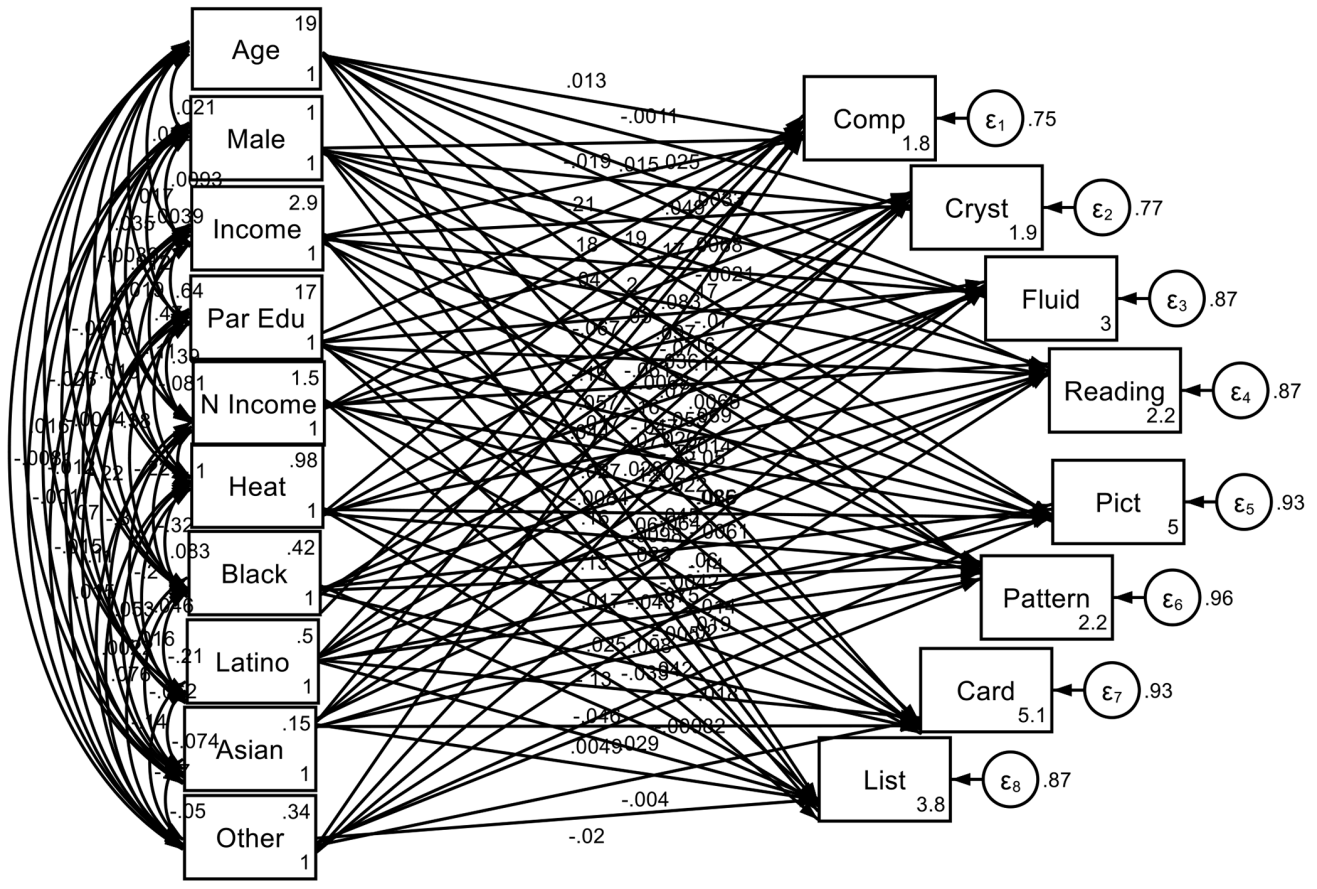
Summary of Structural Equation Modeling (SEM)

As shown by the Table 2 and Figure 1, the analysis revealed a significant negative association between heat exposure and total composite (B=−0.067, p<0.001), crystalized (B=−0.061, p<0.001), fluid composite (B=−0.053, p<0.001), reading (B=−0.050, p<0.001), picture vocabulary (B=−0.025, p=0.008), pattern recognition (B=−0.064, p<0.001), card sorting (B=−0.043, p<0.001), and list recall (B=−0.025, p=0.006) scores.

Total family income was positively associated with total composite (B=0.212, p<0.001), crystalized (B=0.189, p<0.001), fluid composite (B=0.166, p<0.001), reading (B=0.173, p<0.001), and list recall (B=0.161, p<0.001) scores. Parental education was also positively related to total composite (B=0.178, p<0.001), crystalized (B=0.201, p<0.001), fluid composite (B=0.097, p<0.001), reading (B=0.156, p<0.001), and list recall (B=0.134, p<0.001) scores.

Age was positively associated with fluid composite (B=0.025, p=0.005) and pattern recognition (B=0.083, p<0.001) scores. Gender (male) was negatively associated with total composite (B=−0.019, p=0.017), fluid composite (B=−0.049, p<0.001), and pattern recognition (B=−0.070, p<0.001) scores but positively associated with list recall (B=0.027, p=0.002).

Race/ethnicity demonstrated significant associations with cognitive outcomes. Black children exhibited lower total composite (B=−0.186, p<0.001), fluid composite (B=−0.147, p<0.001), and list recall (B=−0.129, p<0.001) scores. Latino children also had lower scores across domains, while Asian children showed higher scores in reading (B=0.060, p<0.001).

## 4. Discussion

We studied the effect of extreme heat exposure on various domains of cognitive function in children and adolescents. Leveraging data from the Adolescent Brain Cognitive Development (ABCD) study [16–25], we tested the adjusted effects of extreme heat exposure on various domains of cognitive function. Additionally, we adjusted for the effects of socio-demographic factors such as race/ethnicity, family and neighborhood SES, that may contribute to disparities in extreme heat exposure and cognitive outcomes. In other words, we explored whether heat exposure might affect cognitive development of children as a part of the influence of environmental stressors on children’s cognitive health.

According to our analysis, strongest adjusted effects were for pattern recognition (B=−0.064, p<0.001) and reading ability (B=−0.050, p<0.001) that belong to the learning domain, total composite (B=−0.067, p<0.001), fluid composite (B=−0.053, p<0.001), and crystallized intelligence (B=−0.061, p<0.001) that belong to General Cognitive Ability followed by. Weaker adjusted effects were found on list recall (B=−0.025, p=0.006) and card sorting (B=−0.043, p<0.001) (memory domain), and picture vocabulary (B=−0.025, p=0.008) (general cognitive ability) [49].

Our prior research [49] found that Black families, households with lower socioeconomic status (SES), and children residing in economically disadvantaged neighborhoods faced higher exposure to extreme heat. This environmental stressor was strongly linked to reduced cognitive function in children, and the association persisted even after accounting for socio-demographic variables. The findings suggest that extreme heat exposure may contribute to lower cognitive performance, with the most pronounced effects observed among marginalized and socioeconomically disadvantaged groups.

In that study, cognitive function was modeled as a latent construct encompassing all cognitive domains collectively. However, our findings indicate that the effects of extreme heat exposure are most pronounced for learning and general cognitive ability, while they are weaker for memory [49].

Extreme heat exposure has been linked to increased substance use, reduced cognitive function, earlier onset of puberty, and a potential rise in delinquent behaviors. Notably, many of these effects persist independently of the socioeconomic disadvantages associated with extreme heat exposure [49, 52, 53]. Therefore, it is crucial to account for confounding factors such as race/ethnicity, family SES, neighborhood SES, age, and sex, all of which were controlled for in this analysis.

In that study, cognitive function was assessed as a latent construct encompassing multiple cognitive domains. However, our findings suggest that extreme heat exposure has the strongest impact on learning and overall cognitive ability, whereas its effect on memory is comparatively weaker [49].

Exposure to extreme heat has also been associated with higher rates of substance use, declines in cognitive function, earlier puberty onset, and a possible increase in delinquent behaviors. Importantly, many of these outcomes appear to persist even when controlling for the socioeconomic disadvantages linked to extreme heat exposure [49, 52, 53]. As a result, it is essential to adjust for potential confounding variables, including race/ethnicity, family and neighborhood SES, age, and sex, all of which were accounted for in this analysis.

Our findings from a nationally representative sample of the ABCD study indicate a significant association between exposure to extreme heat and cognitive function, with disparities observed among children based on race, SES, and financial difficulty. Specifically, Black children, those living in lower SES households, and those residing in economically disadvantaged neighborhoods faced higher exposure to extreme heat. This disproportionate exposure is concerning given the association between extreme heat and diminished cognitive function, including impairments in executive function, memory, and problem-solving. These findings suggest that children already disadvantaged by systemic inequities are also disproportionately affected by environmental stressors, further exacerbating developmental disparities.

A study examined the relationship between extreme heat exposure and delinquent behaviors in children, utilizing ABCD data at baseline. It also investigated potential mediators of this association, including neighborhood SES, puberty, peer deviance, and financial difficulties. SEM were used to explore the link between extreme heat exposure (predictor) and delinquency (outcome). Mediators assessed in the analysis were neighborhood SES, puberty, peer deviance, and financial difficulties. In the total of 11,878 children who were included in the analysis, higher extreme heat exposure was significantly associated with lower cognitive function in children. Children experiencing greater heat exposure were higher delinquent behaviors. They tended to be Black, live in economically disadvantaged neighborhoods, face financial difficulties, and exhibit more advanced puberty status. Authors concluded that the group most affected by extreme heat was disproportionately economically and socially disadvantaged. This study highlights that children already burdened by socio-economic challenges are more vulnerable to the cognitive effects of extreme heat exposure. These findings underscore the importance of targeted interventions to mitigate the impacts of heat exposure and address the underlying disparities. Future research should take advantage of the ABCD longitudinal design. We also need to evaluate the effectiveness of potential environmental interventions that may mitigate the cognitive and developmental risks associated with extreme heat [48].

### Mechanisms Linking Heat Exposure and Cognitive Outcomes

For Black children, the higher exposure to extreme heat can be attributed to historical and systemic factors. The legacy of slavery has concentrated Black populations in southern states that experience more frequent and intense heat waves [30–32]. These regions often have higher rates of poverty and less infrastructure to mitigate the effects of extreme heat [33, 34]. Residential segregation and systemic racism have contributed to Black communities residing in areas with fewer resources, such as limited access to air conditioning, cooling centers, and green spaces [35]. Urban areas with high Black populations are also more likely to experience the urban heat island effect, driven by population density, pollution, and limited vegetation, which further intensifies heat exposure [36, 37].

Children from low-SES families face additional vulnerabilities due to limited access to resources for managing extreme heat [38]. Substandard housing conditions, inadequate insulation, and lack of air conditioning are common in economically disadvantaged households [39]. The financial burden of maintaining cooling systems often makes such measures inaccessible to low-income families [40–42]. This financial strain can exacerbate stress levels, which in turn may impair cognitive function, particularly in domains like attention and executive functioning.

Neighborhood-level SES further compounds the effects of heat exposure. Economically disadvantaged neighborhoods often lack green spaces, have poor infrastructure, and exhibit higher levels of pollution, all of which amplify the adverse effects of heat [44]. Additionally, the absence of community resources such as parks, public pools, and cooling centers leaves children in these areas with few options for mitigating heat exposure. Chronic exposure to such conditions can result in prolonged stress, negatively affecting children’s cognitive development and academic performance.

### Cognitive Implications of Extreme Heat Exposure

Extreme heat can directly and indirectly affect cognitive function through several pathways. Physiological stress responses to heat, such as increased heart rate and dehydration, can impair cognitive processes, including memory and problem-solving. Moreover, chronic heat exposure may contribute to mental health challenges, such as anxiety and depression, which are known to influence cognitive functioning. The discomfort, exhaustion, and irritability caused by extreme heat can further disrupt concentration and decision-making, contributing to poorer cognitive performance across various domains.

The social environment also plays a role. Children exposed to extreme heat may spend more time in unstructured or unsafe outdoor environments, leading to decreased opportunities for cognitive stimulation. Additionally, the cumulative stress of living in heat-vulnerable communities may impact parenting practices, reducing the cognitive and emotional support available to children, thereby exacerbating disparities in cognitive outcomes.

### 4.1. Implications

The findings of this study may have critical implications for research, public health, as well as public policy. First, targeted interventions are essential to reduce the disproportionate exposure of vulnerable populations to extreme heat. Improving housing infrastructure, increasing access to cooling centers, and enhancing urban greening initiatives can help mitigate the cognitive impacts of heat exposure. Schools should also implement measures to ensure children and adolescents have access to environments that have air conditions that can shield them from excessive heat during school activities.

In addition, community-based programs that provide cognitive enrichment activities in safe, climate-controlled spaces can play a crucial role in mitigating the adverse effects of heat on child development. Policymakers must also consider the intersection of environmental and social inequities, focusing on reducing systemic barriers that leave certain populations more vulnerable to climate-related stressors.

### 4.2. Future Research

Future research should prioritize longitudinal studies to explore the cumulative impact of extreme heat exposure on cognitive development over time. Such studies would allow for a more detailed understanding of how repeated exposure to heat waves affects other cognitive domains, including working memory, executive function, and processing speed.

Moreover, additional research should examine the combined, joint, and multiplicative effects of extreme heat exposure and other social and environmental stressors, such as poverty, air pollution, and noise, on cognitive development of children. Understanding these interactions can provide a more comprehensive view of the factors influencing cognitive health in children. Additionally, studies should explore geographic variations in heat exposure and their relationship with cognitive development, as regional differences may reveal specific vulnerabilities and guide tailored interventions.

Intervention-focused research is also critical. Evaluating the effectiveness of cooling centers, urban greening programs, and educational initiatives in improving cognitive outcomes can inform scalable policy solutions. Finally, investigating the role of schools in promoting heat safety practices and mitigating the cognitive impacts of heat exposure can provide actionable insights for educators and policymakers.

### 4.3. Limitations

This investigation had a few methodological limitations that should be noted. The reliance on self-reported measures of exposure and SES introduces the possibility of reporting bias. Furthermore, the cross-sectional design limits the ability to establish causal relationships between heat exposure and cognitive outcomes. Future studies employing longitudinal designs could address these limitations by tracking changes in cognitive performance over time and examining the temporal relationships between heat exposure and cognitive function.

Additionally, while the study accounted for several confounders, other factors, such as parental involvement, access to educational resources, and individual temperament, may also influence the observed relationships. Expanding the range of control variables in future research could help isolate the specific effects of heat exposure on cognitive outcomes.

## 5. Conclusion

This study documented the effects of extreme heat exposure and lower learning, general cognitive ability, and memory in children and adolescents. Vulnerable groups, including Black children, those from lower SES families, and those living in economically disadvantaged neighborhoods, experience disproportionately high heat exposure, exacerbating cognitive disparities. There is an urgent need for targeted policies and interventions that address the compound risks posed by environmental inequities and climate change. As climate change intensifies, proactive measures to mitigate the cognitive and developmental impacts of extreme heat are essential for promoting equity and safeguarding the well-being of all children.

## Figures and Tables

**Table 1.** NIH Toolbox to measure cognitive function represents memory, learning, and general cognitive ability.

**Memory**
• **List Recall.** Directly assesses memory, particularly episodic memory, by testing the ability to recall items from a list.
• **Card Sorting.** While primarily assessing cognitive flexibility and executive function, it also involves working memory to keep track of sorting rules.
**Learning**
• **Pattern Recognition**. Involves the ability to identify and learn patterns, which is a key component of learning and problem-solving.
• **Reading Ability.** Involves learned skills related to language processing and comprehension, indicating prior learning rather than innate ability.
**General Cognitive Ability (G)**
• **Total Composite.** Likely an overall measure of cognitive function, combining multiple subdomains.
• **Fluid Composite.** Measures fluid intelligence (problem-solving and reasoning ability independent of acquired knowledge).
• **Crystallized Intelligence.** Represents accumulated knowledge and verbal skills, heavily influenced by education and experience.
• **Picture Vocabulary.** Taps into crystallized intelligence, as it reflects learned vocabulary and semantic knowledge.

**Table 2.** Summary of Structural Equation Modeling (SEM)

Independent Variable		Dependent Variable	B	SE	95% CI		p
Heat Exposure	→	Total Composite	−0.067	0.009	−0.084	−0.050	< 0.001
Age (Years)	→	Total Composite	0.013	0.008	−0.003	0.029	0.108
Gender (Male)	→	Total Composite	−0.019	0.008	−0.035	−0.004	0.017
Total Family Income	→	Total Composite	0.212	0.012	0.188	0.235	< 0.001
Education Years	→	Total Composite	0.178	0.011	0.157	0.200	< 0.001
Neighborhood Income / 50000	→	Total Composite	0.040	0.010	0.021	0.058	< 0.001
Race / Ethnicity (Black)	→	Total Composite	−0.186	0.010	−0.206	−0.167	< 0.001
Race / Ethnicity (Latino)	→	Total Composite	−0.057	0.009	−0.076	−0.039	< 0.001
Race / Ethnicity (Asian)	→	Total Composite	0.044	0.008	0.028	0.060	< 0.001
Race / Ethnicity (Other)	→	Total Composite	−0.008	0.009	−0.025	0.008	0.324
Intercept	→	Total Composite	1.841	0.232	1.386	2.295	< 0.001
							
Heat Exposure	→	Crystal	−0.061	0.009	−0.078	−0.044	< 0.001
Age (Years)	→	Crystal	−0.001	0.008	−0.017	0.015	0.893
Gender (Male)	→	Crystal	0.015	0.008	−0.001	0.031	0.072
Total Family Income	→	Crystal	0.189	0.012	0.166	0.213	< 0.001
Education Years	→	Crystal	0.201	0.011	0.179	0.222	< 0.001
Neighborhood Income / 50000	→	Crystal	0.030	0.010	0.011	0.049	0.002
Race / Ethnicity (Black)	→	Crystal	−0.165	0.010	−0.184	−0.146	< 0.001
Race / Ethnicity (Latino)	→	Crystal	−0.073	0.009	−0.091	−0.054	< 0.001
Race / Ethnicity (Asian)	→	Crystal	0.029	0.008	0.013	0.045	< 0.001
Race / Ethnicity (Other)	→	Crystal	−0.010	0.009	−0.027	0.007	0.255
Intercept	→	Crystal	1.945	0.234	1.485	2.404	< 0.001
							
Heat Exposure	→	Fluid Composite	−0.053	0.009	−0.071	−0.035	< 0.001
Age (Years)	→	Fluid Composite	0.025	0.009	0.007	0.042	0.005
Gender (Male)	→	Fluid Composite	−0.049	0.009	−0.066	−0.032	< 0.001
Total Family Income	→	Fluid Composite	0.166	0.013	0.141	0.191	< 0.001
Education Years	→	Fluid Composite	0.097	0.012	0.074	0.120	< 0.001
Neighborhood Income / 50000	→	Fluid Composite	0.036	0.010	0.015	0.056	0.001
Race / Ethnicity (Black)	→	Fluid Composite	−0.147	0.011	−0.168	−0.127	< 0.001
Race / Ethnicity (Latino)	→	Fluid Composite	−0.022	0.010	−0.042	−0.002	0.028
Race / Ethnicity (Asian)	→	Fluid Composite	0.045	0.009	0.028	0.062	< 0.001
Race / Ethnicity (Other)	→	Fluid Composite	−0.004	0.009	−0.022	0.014	0.649
Intercept	→	Fluid Composite	3.043	0.250	2.553	3.533	< 0.001
							
Heat Exposure	→	Reading	−0.050	0.009	−0.068	−0.032	< 0.001
Age (Years)	→	Reading	0.003	0.009	−0.014	0.020	0.702
Gender (Male)	→	Reading	0.007	0.009	−0.010	0.024	0.434
Total Family Income	→	Reading	0.173	0.013	0.148	0.198	< 0.001
Education Years	→	Reading	0.156	0.012	0.133	0.179	< 0.001
Neighborhood Income / 50000	→	Reading	0.007	0.010	−0.013	0.026	0.521
Race / Ethnicity (Black)	→	Reading	−0.086	0.010	−0.107	−0.066	< 0.001
Race / Ethnicity (Latino)	→	Reading	−0.006	0.010	−0.026	0.014	0.546
Race / Ethnicity (Asian)	→	Reading	0.060	0.009	0.043	0.077	< 0.001
Race / Ethnicity (Other)	→	Reading	0.019	0.009	0.001	0.037	0.035
Intercept	→	Reading	2.219	0.248	1.734	2.704	< 0.001
							
Heat Exposure	→	Picture	−0.025	0.009	−0.044	−0.007	0.008
Age (Years)	→	Picture	−0.002	0.009	−0.020	0.015	0.817
Gender (Male)	→	Picture	−0.070	0.009	−0.087	−0.053	< 0.001
Total Family Income	→	Picture	0.105	0.013	0.079	0.131	< 0.001
Education Years	→	Picture	0.069	0.012	0.046	0.093	< 0.001
Neighborhood Income / 50000	→	Picture	0.014	0.011	−0.006	0.035	0.176
Race / Ethnicity (Black)	→	Picture	−0.137	0.011	−0.158	−0.116	< 0.001
Race / Ethnicity (Latino)	→	Picture	−0.014	0.010	−0.034	0.006	0.179
Race / Ethnicity (Asian)	→	Picture	0.020	0.009	0.003	0.038	0.023
Race / Ethnicity (Other)	→	Picture	−0.018	0.009	−0.037	0.000	0.050
Intercept	→	Picture	4.974	0.258	4.469	5.478	< 0.001
							
Heat Exposure	→	Pattern	−0.064	0.010	−0.082	−0.045	< 0.001
Age (Years)	→	Pattern	0.083	0.009	0.066	0.101	< 0.001
Gender (Male)	→	Pattern	−0.070	0.009	−0.088	−0.052	< 0.001
Total Family Income	→	Pattern	0.070	0.013	0.043	0.096	< 0.001
Education Years	→	Pattern	0.026	0.012	0.002	0.050	0.033
Neighborhood Income / 50000	→	Pattern	0.020	0.011	−0.001	0.041	0.056
Race / Ethnicity (Black)	→	Pattern	−0.075	0.011	−0.096	−0.053	< 0.001
Race / Ethnicity (Latino)	→	Pattern	−0.005	0.011	−0.026	0.015	0.615
Race / Ethnicity (Asian)	→	Pattern	0.042	0.009	0.025	0.060	< 0.001
Race / Ethnicity (Other)	→	Pattern	−0.001	0.010	−0.020	0.018	0.931
Intercept	→	Pattern	2.182	0.259	1.674	2.690	< 0.001
							
Heat Exposure	→	Card Sort	−0.043	0.010	−0.062	−0.024	< 0.001
Age (Years)	→	Card Sort	−0.007	0.009	−0.024	0.011	0.447
Gender (Male)	→	Card Sort	−0.047	0.009	−0.065	−0.030	< 0.001
Total Family Income	→	Card Sort	0.115	0.013	0.090	0.141	< 0.001
Education Years	→	Card Sort	0.067	0.012	0.043	0.091	< 0.001
Neighborhood Income / 50000	→	Card Sort	0.023	0.011	0.002	0.044	0.029
Race / Ethnicity (Black)	→	Card Sort	−0.098	0.011	−0.120	−0.077	< 0.001
Race / Ethnicity (Latino)	→	Card Sort	−0.033	0.010	−0.053	−0.013	0.001
Race / Ethnicity (Asian)	→	Card Sort	0.029	0.009	0.011	0.046	0.001
Race / Ethnicity (Other)	→	Card Sort	−0.004	0.009	−0.022	0.014	0.672
Intercept	→	Card Sort	5.147	0.258	4.641	5.653	< 0.001
							
Heat Exposure	→	List	−0.025	0.009	−0.043	−0.007	0.006
Age (Years)	→	List	0.017	0.009	0.000	0.034	0.055
Gender (Male)	→	List	0.027	0.009	0.010	0.044	0.002
Total Family Income	→	List	0.161	0.013	0.136	0.186	< 0.001
Education Years	→	List	0.134	0.012	0.111	0.157	< 0.001
Neighborhood Income / 50000	→	List	0.017	0.010	−0.003	0.037	0.090
Race / Ethnicity (Black)	→	List	−0.129	0.010	−0.150	−0.109	< 0.001
Race / Ethnicity (Latino)	→	List	−0.046	0.010	−0.065	−0.026	< 0.001
Race / Ethnicity (Asian)	→	List	0.005	0.009	−0.012	0.022	0.572
Race / Ethnicity (Other)	→	List	−0.020	0.009	−0.038	−0.002	0.030
Intercept	→	List	3.801	0.251	3.310	4.292	< 0.001
